# The Restoration of Passive Rotational Tibio-Femoral Laxity after Anterior Cruciate Ligament Reconstruction

**DOI:** 10.1371/journal.pone.0159600

**Published:** 2016-07-28

**Authors:** Philippe Moewis, Georg N. Duda, Tobias Jung, Markus O. Heller, Heide Boeth, Bart Kaptein, William R. Taylor

**Affiliations:** 1 Julius Wolff Institute, Charité-Universitätsmedizin Berlin, Berlin, Germany; 2 Knee Surgery and Sports Traumatology, Center for Musculoskeletal Surgery, Charité-Universitätsmedizin Berlin, Berlin, Germany; 3 Bioengineering Research Group, University of Southhampton, Southhampton, United Kingdom; 4 Department of Orthopaedic Surgery, Biomechanics and Imaging Group, Leiden University Medical Center, Leiden, Netherlands; 5 Department of Health Sciences and Technology, Institute for Biomechanics, ETH Zürich, Zürich, Switzerland; Queen's University, CANADA

## Abstract

While the anterior cruciate ligament (ACL) is considered one of the most important ligaments for providing knee joint stability, its influence on rotational laxity is not fully understood and its role in resisting rotation at different flexion angles *in vivo* remains unknown. In this prospective study, we investigated the relationship between *in vivo* passive axial rotational laxity and knee flexion angle, as well as how they were altered with ACL injury and reconstruction. A rotometer device was developed to assess knee joint rotational laxity under controlled passive testing. An axial torque of ±2.5Nm was applied to the knee while synchronised fluoroscopic images of the tibia and femur allowed axial rotation of the bones to be accurately determined. Passive rotational laxity tests were completed in 9 patients with an untreated ACL injury and compared to measurements at 3 and 12 months after anatomical single bundle ACL reconstruction, as well as to the contralateral controls. Significant differences in rotational laxity were found between the injured and the healthy contralateral knees with internal rotation values of 8.7°±4.0° and 3.7°±1.4° (*p* = 0.003) at 30° of flexion and 9.3°±2.6° and 4.0°±2.0° (*p* = 0.001) at 90° respectively. After 3 months, the rotational laxity remained similar to the injured condition, and significantly different to the healthy knees. However, after 12 months, a considerable reduction of rotational laxity was observed towards the levels of the contralateral controls. The significantly greater laxity observed at both knee flexion angles after 3 months (but not at 12 months), suggests an initial lack of post-operative rotational stability, possibly due to reduced mechanical properties or fixation stability of the graft tissue. After 12 months, reduced levels of rotational laxity compared with the injured and 3 month conditions, both internally and externally, suggests progressive rotational stability of the reconstruction with time.

## Introduction

Although a natural amount of passive joint laxity exists within healthy joints, excessive laxity is often a direct consequence of failure of one or more musculoskeletal structures, particularly after traumatic injury [1]. In the knee, passive laxity is primarily governed by the ligaments. While the primary function of the anterior cruciate ligament (ACL) is to stabilize against excessive tibial translation relative to the femur [2], it is also thought to play a secondary role in controlling axial rotation, particularly internally, and hence contribute towards rotational stabilization of the knee joint [3]. As a result, injuries of the ACL have a direct repercussion on knee joint laxity and kinematics, resulting in both increased anterior-posterior (A-P) tibial displacement and axial rotation [4].

Although patients with ACL rupture present passive instability or excessive laxity, some individuals are able to actively stabilize their knees during activities of daily living [5]. As a result of the altered kinematics, together with the associated prevalence of degenerative changes in the longer term [6], reconstruction of the ACL becomes the primary option for restoring normal function and kinematics of the injured knee. However, it is plausible that rotational instability after ACL reconstruction could be a contributing factor towards graft failure [7], and might also play a role in the initiation of biological and mechano-degenerative processes such as osteoarthritis (OA) [8–10]. The quest for effective reconstruction of knee rotational stability therefore represents a key challenge for surgeons [11], where an understanding of rotational laxity in healthy knees, as well as after ACL reconstruction, is clearly required.

Typically, rotational laxity is assessed in the clinic using the pivot shift test [12], however this test lacks objectivity and is dependent upon the examiner’s experience [13, 14]. Although a range of devices for analysing rotational laxity have been employed, including goniometers [15], electromagnetic sensors [16, 17], light-emitting diode (LED)-markers [18], electronic sensors [19], inclinometers [20], and magnetic resonance imaging (MRI) [21, 22], these approaches are generally subject to soft tissue artefact (and thus inaccurate or over-estimate the real skeletal rotation [23]) or may be limited due to the extended periods of time required for image capture. Here, MRI approaches have been used to good effect in the comparison of axial rotation between ACL reconstructed and healthy knees, and have demonstrated a post-reconstruction reduction in the axial rotational range of motion (RoM) [22], albeit only at 15° of knee flexion. On the other hand, a study using electronic sensors attached to the skin to assess tibio-femoral motion reported no significant differences between the ACL reconstructed and healthy contralateral knees [24]. These reports suggest that the outcome of ACL reconstruction and its effects on rotational stability–or indeed the approach used to assess the laxity–remain controversial. More recently, navigation systems were used to examine differences in internal rotation (IR) and external rotation (ER) intra-operatively [25, 26]; however the application of torque was surgeon dependent, which could have influenced the results, and the technique did not allow for multiple follow-up assessments. Importantly, however, the influence of knee flexion as well as post-operative recovery is not well understood, but could provide an improved insight into the influence of ACL reconstruction on joint rotational laxity.

One approach that is known to allow rotation of skeletal structures to be determined accurately is videofluoroscopy, which is an established technique to assess dynamic activities *in vivo*, and has been used in the kinematic assessment of implanted components, as well as for examining the motion of bone segments [23, 27–30]. Application of this technique, together with a device for the objective and controlled rotation of the knee joint, could help towards understanding the influence of the ACL on rotational laxity of the knee joint and at a range of knee joint flexion angles.

In this pilot study, we therefore aimed to prospectively investigate *in vivo* passive axial rotational laxity of the tibio-femoral joint, and at different knee flexion angles. Furthermore, by performing measurements pre-operatively, as well as at 3 and 12 months post-operatively, we aimed to objectively assess the changes in passive rotational laxity that occurs after ACL reconstruction.

## Materials and Methods

### Ethics statement

The study was approved by the local ethics committee (Ethikkommission, Campus Charité Mitte, Charité-Universitätsmedizin Berlin) and all subjects provided written informed consent prior to participation (Approval Number: EA1/167/08).

### Subjects

Thirteen subjects (age: 30 ± 8 years, BMI: 25 ± 3, **♂**: 9, ♀: 4) with one-sided rupture of the ACL were recruited within six months. Recruitment took place at the Center for Musculoskeletal Surgery, Charité—Universitätsmedizin in Berlin, Germany. The diagnosis was first conducted clinically and confirmed by MRI. Subjects with other concomitant injuries were excluded.

All subjects underwent computer tomography (CT) scanning (Siemens Sensation 64, 512 x 512 image matrix, in plane resolution 0.4 mm x 0.4 mm, slice thickness 1 mm) of the injured as well as the contralateral knees, which were used as controls. All testing of subjects involved in this study were performed in accordance with the Declaration of Helsinki.

Internal and external rotational laxity (as described below) were measured at three time points; ACL injured (one to three months after injury), 3 months post-ACL-reconstruction, and 12 months post-ACL-reconstruction. In addition, the healthy contralateral knees were tested during the first measurement session. Four subjects did not complete the 12 month follow-up measurement; two subjects had moved away from the area and withdrew their consent to participate in the study, and two had suffered a re-rupture of the ACL and needed further operative reconstruction. As a consequence, results for only the 9 subjects that completed all measurements are reported in the results section.

### ACL reconstruction procedure

In all patients, single bundle ACL reconstruction with autologous semitendinosus implant grafting was conducted using a hybrid technique that used an endobutton and bio-resorbable interference screw in each of the tibia and femur [31]. This approach is able to prevent the requirement for oversized screws as well as avoid possible bungeeing of the graft across the joint gap, while still maintaining many of the advantages of more standard fixation techniques. The length of the extracted tendon was approximately 26 cm, and was chosen in order to achieve four strands, which were sutured together, leaving a transplant graft of almost 65 mm in length. The procedure aimed to reconstruct the anatomical configuration of the ACL [31]. For the femoral tunnel drilling, the knee joint was flexed at 120° to achieve a 10 or 2 o’clock position, hence also providing good accessibility to the anatomical ACL origin. To avoid perforation of the lateral femoral cortex, a maximal screw drilling depth of between 30 and 40 mm was targeted, which accounted for small and large knee joints respectively [31]. To drill the tibial screw tunnel, the knee was flexed between 50 and 70°, where an optimal view of the tibial ACL insertion could be achieved. A minimum of 18 mm of the screw was then introduced into the femoral tunnel, while 20–22 mm was introduced into the tibial tunnel under arthroscopic control.

All patients underwent the same rehabilitation protocol. Depending on the patient’s recovery, jogging was allowed as early as 3 months with a return to sporting activity after 6 months [31]. As part of the standard clinical examination, passive A-P translation was also assessed using a KT-1000 arthrometer with an applied anterior tibial force of 133N.

### Experimental set-up

**Fig 1:**
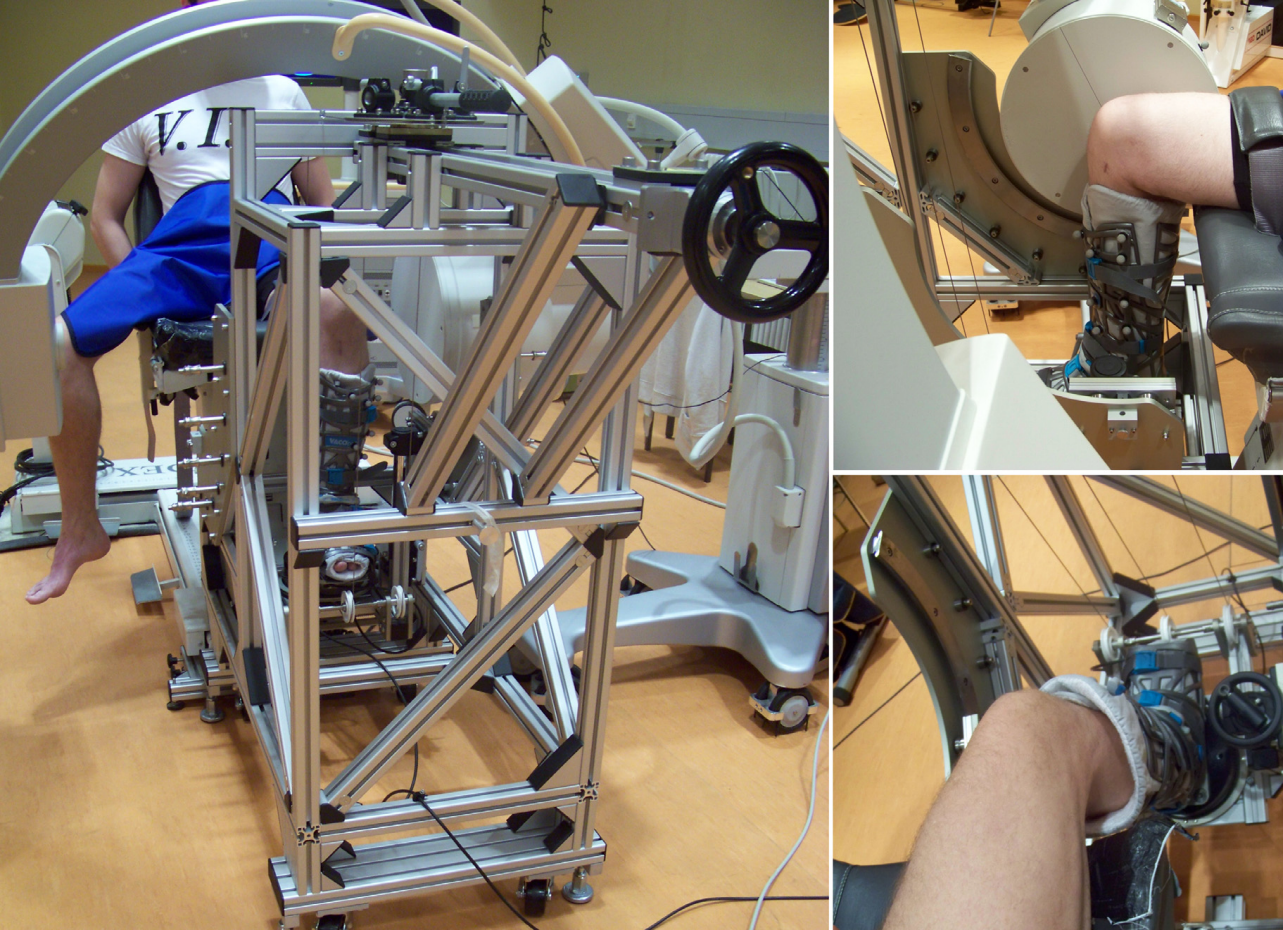
**Left: Measurement set-up showing a subject seated and positioned within the knee Rotometer, together with the fluoroscopic device. Top-right: Patient´s shank in the Vacoped boot and knee centred in front of the image intensifier at 90° flexion. Bottom-right: Visualization of the patient´s shank in the Vacoped boot and the attached force transducer. No artificial constraints were applied to the knee**.

A system for the accurate and objective measurement of passive rotational laxity of the knee (knee rotometer) was constructed (Fig 1, left) and certified for use in human experiments by CERT (Berlin Certification authority; certification number: Z-11-131-MP). The device allowed internal and external rotation of the tibio-femoral joint to be performed at a range of knee flexion angles from 0° to 90°. To ensure as far as possible that all rotation occurred at the tibio-femoral joint (rather than at the ankle joint), the foot, ankle and shank were strapped into a Vacoped boot (OPED GmbH, Oberlaindern, Germany). No additional constraints were applied to the knee itself (Fig 1, top and bottom right). The boot was connected to a rotating plate that was attached to a 6-degree of freedom force transducer (ATI Industrial Automation, Apex, USA; accuracy 0.06Nm). An axial torque was then applied manually using an application lever. This axial torque was transferred via a cable pulley mechanism to the rotating plate, to which the Vacoped boot was attached, subsequently rotating the tibia relative to the femur.

Over-rotation of the tibia was avoided by an audible feedback signal, which indicated the limits of the applied torque measured by the force transducer, controlled using a Labview (National Instruments, Austin, USA) software application. In addition, a physical stop ensured that no axial rotation beyond ±40° was possible. During each measurement, the subject was positioned in a comfortable, safe and adjustable seat (Biodex system 4, USA).

Throughout the measurements, the thigh and waist were both strapped firmly to the seat to minimize movement of the femur and pelvis. The resulting constraints ensured that almost no global knee anterior-posterior movement and only minimal medial-lateral translation of the entire knee joint was possible–however, even if small movement was possible, the fluoroscopic evaluation of the tibia and femur (as described below) ensured that only the relative rotation of the skeletal structures was determined.

### Testing procedures

Two investigators worked as a team to perform all measurements using consistent procedures. While axial torque values ranging from 2 to 15 Nm have been reported in the literature [32–37] (with higher torques only applied during *in vitro* tests or under anaesthetic conditions), values above 3Nm were precluded by our clinicians due to potential damage to freshly reconstructed grafts. As a result, a maximum internal and external torque of 2.5 Nm was used in this study. This torque value was set as the audible threshold to prevent over-rotation of the joint during testing. The subjects were instructed to relax their leg muscles to allow the examiner to manually rotate the tibia without resistance due to muscular activation–here, any muscular activity could be clearly seen in perturbations to the torque output, whereupon the cycle was re-measured. Beginning at the comfortable resting position of the shank, the measurement of a complete axial rotation cycle consisted of internal rotation, up to the maximum 2.5 Nm torque internally, followed by external rotation of the tibia up to the same torque value externally, and returning to the resting position. Simultaneously, relative rotation of the tibia and femur was assessed fluoroscopically.

### Fluoroscopic analysis and quantification of skeletal tibio-femoral rotation

A C-arm fluoroscope (Pulsera BV, Philips) was positioned at the level of the knee with the centre of the knee beside the centre of the image intensifier (Fig 1). Prior to each measurement, the fluoroscopic system was calibrated in order to correct for image distortion using a specially designed Perspex calibration box (BAAT Engineering B.V. Hengelo) [38]. Fluoroscopic images were collected during the complete axial rotation cycle at a frequency of 3 Hz. The total effective dosage for each measurement, calculated from the dose area product, ranged from 0.002 to 0.0075 mSv. Use of X-rays (CT and Fluoroscopy) on the subjects was approved by the Bundesamt für Strahlungsschutz (Approval Number: Z5-22462/2-2010-003).

A scattered radiation sensor (Silicon Sensor International AG; delay 50 ns) was used to synchronize the torque sensor and the fluoroscopic imaging system. The signal produced by the sensor was continually transferred via a PCI NI card in the torque acquisition Labview program, allowing for synchronisation of the fluoroscopic and axial torque data during post-processing.

3D bone models of each subjects’ tibia and femur were reconstructed from the individual CT datasets using the Amira software suite (Amira, Visage Imaging, Berlin, Germany), and were then registered to the fluoroscopic images to calculate the tibio-femoral rotation using Model-Based RSA software (RSA*core*, Leiden University Medical Center, Leiden, The Netherlands). The pose of the 3D bone models was determined by fitting two sets of outline contours, one from the projection of the bone models and one from the fluoroscopic images, to create the optimal matching scenario for each time point [39]. The accuracy of this procedure has previously been assessed, with rotational errors of up to 1° [40]. Axial rotation of the tibia relative to the femur was then calculated for all fluoroscopic images of knee joint rotation. Since the fluoroscopic technique allowed direct visualization and then reconstruction of both the femur and the tibia in 3D space, the assessment of axial rotation was not susceptible to soft tissue artefact, allowing an accurate determination of the relative tibio-femoral rotation.

### Measurement of Rotational Laxity

Measurements were performed at 30 and 90° of knee flexion. The 30° measurement position was chosen since the ACL is thought to be tensioned without additional stabilization from the other ligaments of the knee [2]. Testing was also performed at 90° due to the lack of congruency between the bone structures and therefore the high dependency upon the soft tissue structures alone for resisting rotational laxity of the knee in general. Measurements at full extension were avoided due to the complex interaction of the screw home mechanism, locking of the joint and tension in the hamstrings, which would likely produce an unclear test outcome [2, 23]. The intra-tester reliability of the procedure to apply rotational torque to the knee joint has been previously assessed using the intra-class correlation coefficient (ICC 3,1) with values of 0.99 and 0.98 for measurements at 30° and 90° of flexion respectively [23].

Torque-rotation curves, constructed from the applied axial torque and the calculated axial rotation from the fluoroscopy, were created for every measurement time point. The peak rotations at ±2.5 Nm were used as a measure of internal and external rotational laxity. To correct for the effect of each subject’s natural knee rotation angle, the neutral reference rotation for each subject was determined as the average angle at which zero resistance to rotation was observed (taking rotation in both the internal and external directions into consideration). These neutral reference positions were then aligned for group-wise analyses.

### Statistical Analysis

Statistical analysis of the torque-rotation data was performed using the SPSS software package (SPSS v23.0, IBM Corp., Armonk, USA). Based on the investigative nature of this study, we report the key results descriptively by providing group means and standard deviations of rotation during the loading phase of the measurement cycle, specifically when the torque first reached 2.5Nm. After testing for normal distribution of the data using the Kolmogorov–Smirnov test, the Student’s T–test was used to compare the joint laxity at the three time points of the injured and reconstructed ACL knees against the healthy contralateral knees. In order to assess the effect of the progressive changes in A-P laxity (assessed using the KT-1000) side-to-side differences were examined using the Student’s T–test. A *p* value < 0.05 was regarded as statistically significant.

## Results

**Fig 2:**
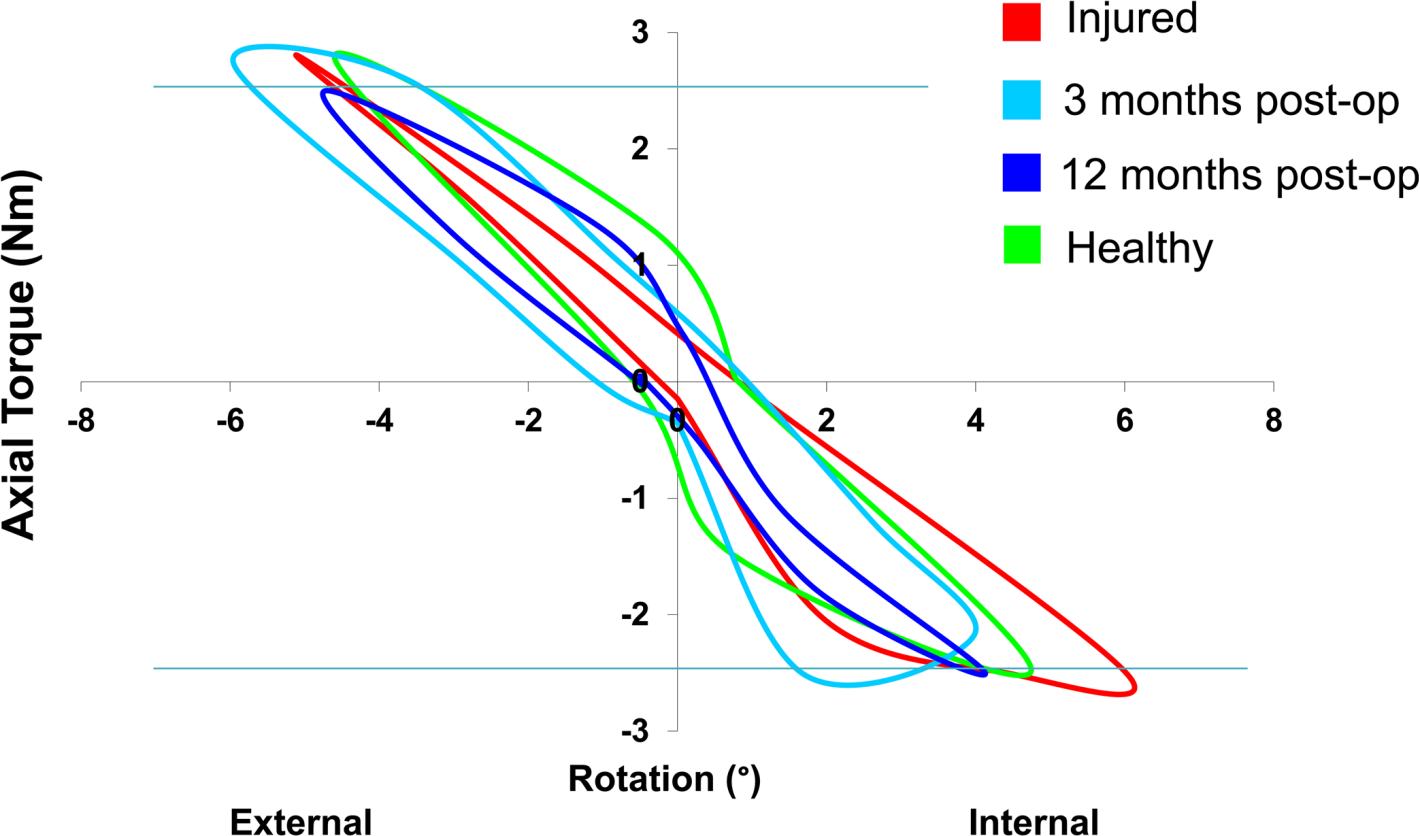
Example of the torque-rotational curves of one patient at the pre-operative (injured), 3 month postoperative and 12 month postoperative time points, as well as the healthy contralateral knee (healthy).

Only low levels of discomfort were communicated by the subjects during testing. Each cycle of internal and external rotation showed a clear hysteresis (shown exemplary at 30° for one subject, Fig 2), with each curve crossing or at least reaching the ±2.5Nm threshold. Although high inter-subject variability was observed in the amount of tibio-femoral rotation, all subjects exhibited similar curves for every test.

**Fig 3:**
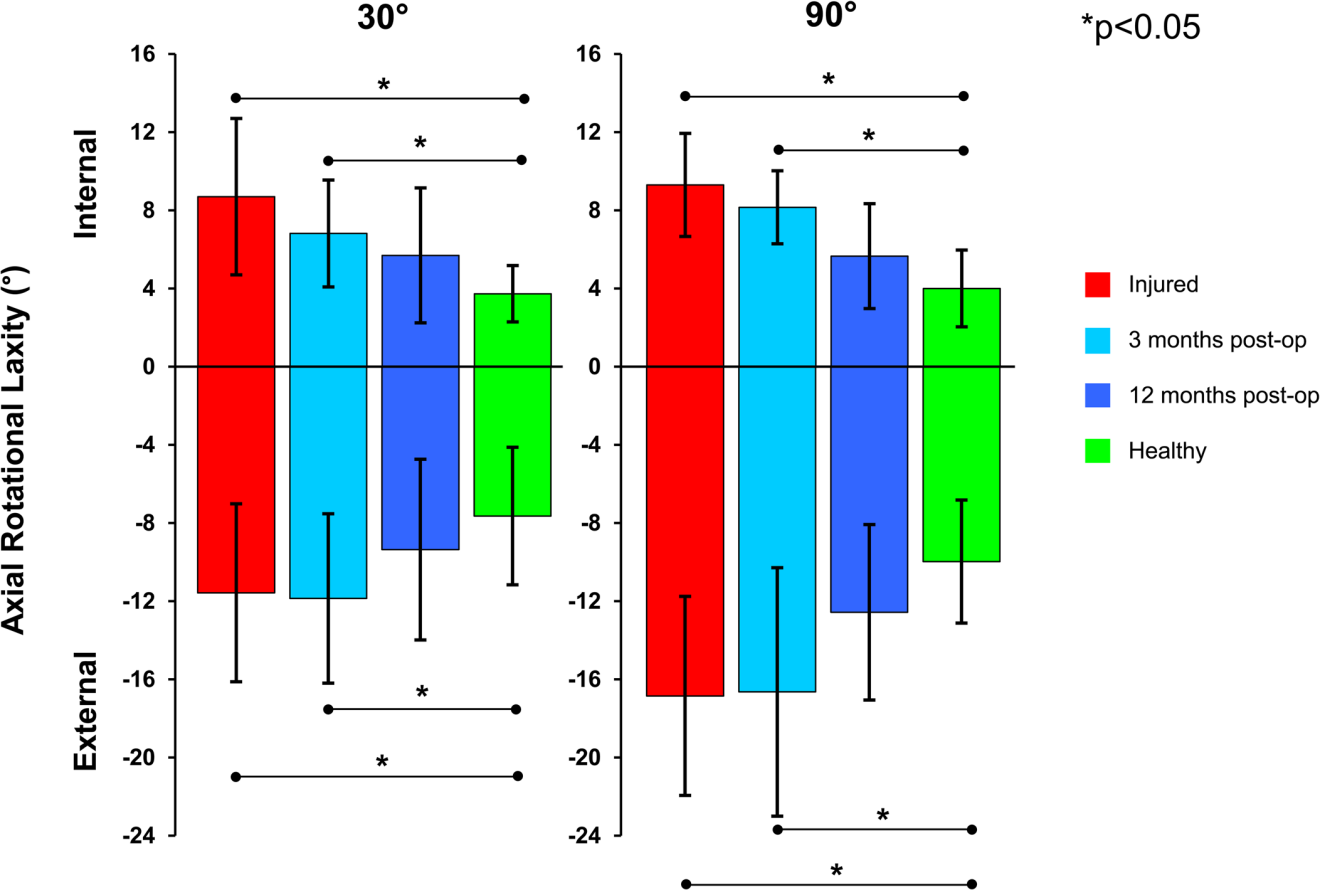
Internal and external rotational laxity of the analysed subjects at all time points compared against the healthy contralateral knee (shown in green) at both 30° and 90° flexion angles.

Significant differences in the internal rotational laxity were found between the ACL injured and the healthy contralateral knees with values of 8.7°±4.0° (mean±SD) and 3.7°±1.4° (*p* = 0.003) at 30° of flexion and 9.3°±2.6° and 4.0°±2.0° (*p* = 0.001) at 90° for the ACL injured and healthy knees respectively. For external rotational laxity, the values were 11.6°±4.5° and 7.6°±3.5° (*p* = 0.004) at 30° and 16.8°±5.1° and 10.0°±3.1° (*p* = 0.005) at 90° for the ACL injured and healthy knees respectively (Fig 3). Three months after ACL reconstruction, a reduction of the internal rotational laxity was observed at both flexion angles, while the values for external rotational laxity were similar. Both internal (*p* = 0.005, *p* = 0.006) and external (*p* = 0.001, *p* = 0.004) laxity remained significantly greater than the healthy contralateral knees at 30° and 90° of joint flexion respectively. From three to twelve months, a further improvement of the rotational stability could be observed, resulting in values comparable with those of the healthy contralateral side. Comparing both flexion angles, higher values of both internal and external rotational laxity and therefore also total axial RoM were observed at 90° of knee flexion, indicating a higher passive rotational laxity at higher flexion.

Anterior-posterior (A-P) translational laxity determined using the KT-1000 arthrometer exhibited a reduction in the side-to-side differences (compared to the healthy contralateral limb) for the injured (3.9mm±1.9mm) versus the 3 month post-operative (1.8mm±2.5mm) time points (*p* = 0.027). A progression in reduction in side-to-side differences was observed (*p* = 0.004) from the 3 month to the 12 month post-operative follow-up (0.4mm±2.0mm).

## Discussion

Excessive laxity of articulating joints is known to lead to degenerative changes of the local cartilage [4, 6]. However, little is known about changes to rotational laxity of the knee joint that occur after ACL rupture, and their progression post-reconstruction. During functional activities, both passive and active structures contribute towards guiding the tibio-femoral kinematics as well as stabilising the knee joint [41, 42], while the passive structures are known to play a key role in restricting the extremes of movement [18]. As such, ligament reconstruction must then target a complete biological and mechanical recovery for full and stable function of the knee joint to be achieved. While assessment of knee joint A-P translational stability is standard in the clinic [41], objective measurements of rotational laxity remain widely missing [43]. Such knowledge can help the success of surgical reconstruction to be monitored, as well as lay the foundations for understanding the restoration of rotational joint stability after surgery.

In the present study, a rotometer device was specially designed to measure the knee joint rotational laxity in an objective manner. Significant differences in rotational laxity were found between the injured and the healthy contralateral knees at 30 and 90° of knee joint flexion. After 3 months, a significant difference in internal rotational laxity compared to the controls was still observed, and the total range of passive motion of the joint (under the externally applied 2.5Nm torque) remained similar to the injured condition. Furthermore, the significantly greater laxity observed at both knee flexion angles after 3 months, but not at 12 months, suggests an initial lack of post-operative stability, possibly due to reduced mechanical properties or fixation stability of the graft tissue.

Although differences in rotational laxity have been observed between ACL resected/deficient and intact knees both *in vitro* [44–48] and *in vivo* [22, 24, 25, 32, 49], these studies lack either applicability or objectivity due to the torque application techniques or the methods for assessing skeletal rotation. In our study, significant differences in the internal rotational laxity were observed *in vivo* between ACL injured and healthy contralateral knees at both the 30° and 90° knee flexion angles tested. At 30°, this result was not entirely unexpected, since the ACL is thought to be mainly responsible for providing passive stabilization of the knee joint at this flexion angle due to laxity of the other supporting ligaments [2]. At 90°, however, two important observations could be made. In healthy knees, the ACL is thought to be moderately relaxed [11, 50–52]. The greater laxity of the healthy knees (compared to the 30° position) was therefore considered reasonable. However, the significantly greater joint laxity of the injured knees (compared to the healthy counterparts at 90°) was somewhat unexpected, and indicates that the ACL might indeed play an important role for joint stability at this flexion angle. Here, the mechanisms for the ACL to provide rotational stability are somewhat unclear, especially for both internal and external rotation, but could be related to the ligament’s ability to pull the joint surfaces together, therefore gaining joint rotational stability through providing pressure between the congruent articulating joint structures. This hypothesis may also partially explain the different stability observed at different flexion angles, where changing tension in the ACL may play a role. Alternatively, it is entirely possible that sub-clinical damage had indeed occurred to further surrounding structures in some of our subjects, thus contributing to an increase in joint laxity at 90°. Further study to confirm our surprising findings and to explain these complex interactions is clearly required.

Although a reduction of the internal rotational laxity was observed after 3 months at both 30° and 90° flexion (Fig 3), there were still significant differences compared with the healthy knees, which indicates a remaining rotational instability even after surgery, and could be related to a decrease of mechanical properties or fixation of the graft tissue [53, 54]. Here, a reduction in the mechanical properties of autologous ligament graft tissue was observed in a sheep model of semitendinosus graft reconstruction of the ACL, implanted in a similar manner to that employed in the present study in humans. In assessing the translational stability of the reconstruction over the course of healing, the authors suggested that the reduction of mechanical stability was a result of the biological remodelling processes. Here, the reorganization of the graft’s extracellular matrix showed a reconstitution of a similar-to-natural fold and a re-vascularization of the graft around the sixth to eighth week due to graft remodelling [54]. This observation was accompanied by changes in elongation of the graft over the first 9 weeks, with some slight improvement after 12 weeks and a reconstitution of mechanical competence up to a year following surgical reconstruction. Although the extent of initial loss of mechanical competence in sheep may not be comparable to humans [53], the findings from the animal experiment could serve to explain the observed laxity following surgical ACL reconstruction at 3 months in humans, as well as the recovery at 12 months. This progressive reduction of laxity with increasing time (no significant differences were observed at 12 months) is supported by literature [22, 24], where no significant differences in rotational stability were observed after single bundle reconstruction using a hamstring auto-graft or a bone-patellar-bone tendon graft after a mean follow-up of 27 months. In agreement with the findings in the rotational parameters, the routine analysis with the KT-1000 showed a progressive reduction in side-to-side differences for the A-P translation of the tibia relative to the femur, hence confirming a general translational and rotational stabilization of the knee joint after ACL reconstruction in our study. However, in line with current clinical experience, the instability observed in our study after 3 months also highlights the importance for patients to undertake and complete rehabilitation programmes, and that the risk of re-rupture when returning to sporting activities at an early time point should not be underestimated [55].

During testing, each subject´s natural tibio-femoral rotation was determined as the rotation at 0Nm torque, using data taken from complete cycles of both internal and external rotation. For the subjects tested here, approximately 6–8° of natural external tibial rotation (relative to the rotometer 0° axis) was observed. From the applied rotation, the results of this study indicate that the total axial RoM was similar between the ACL injured condition and the 3 month post-reconstruction knees, but that the natural rotation angle of the knee was altered by about 1–2°. These data suggest that the ACL is under natural passive tension at both 30° and 90° flexion in order to maintain this small external tibial rotation. After ACL rupture, it seems that this tension is released, resulting in a small internal rotation of the tibia relative to the femur. This concept would be consistent with the idea that the tibio-femoral centre of rotation in the transverse plane is medial of the line of action of the ACL [56–58]. Although these findings remain to be corroborated in further investigations, it is clear that any variation in the centre of rotation–which is thought to also be activity dependent [59]–could alter employment of the ACL.

Although this study was initially planned as an investigative pilot study with 13 subjects recruited, only 9 subjects successfully completed all measurements. However, a *post-hoc* analysis of the data has revealed that 9 subjects would be required to demonstrate a difference between 3-month post-reconstruction laxity and the healthy contralateral knees (with a power above 0.8), suggesting that the study is able to draw statistically relevant conclusions, at least in some areas of the analysis. The authors do acknowledge, however, that while the general findings in this investigation are informative, the fact that 4 subjects could not be measured after 12 months postoperatively represents a weakness in the study.

The results of the present study provide an insight into the influence of the ACL on rotational laxity at different flexion angles. A similar trend in laxity changes over time was observed for internal rotational laxity and RoM at both 30° and 90° flexion angles. However, a higher external rotational laxity, and therewith associated RoM, was observed at 90° of flexion, possibly influenced by the lack of congruency between the bone structures [2, 60]. Increased laxity at 90° flexion is contrary to the results presented by Park and colleagues [18], where a reduction of tibial rotation was observed at higher flexion, in their case 60°. It is important to note, however, that only healthy athletes were examined in the study of Park et al., where methods that could be affected by skin motion artefact were used [23]. Although all of our subjects had a confirmed isolated ACL injury, the possible, but not confirmed, negative influence of this injury on the other passive structures cannot be excluded, further complicating any comparison to the results of Park and co-workers.

Although measurements of coupled A-P translation and axial rotation of the knee joint would be interesting, this study specifically targeted a comprehension of rotational laxity within the knee, and no anterior or posterior forces were applied to the joint. In support of this idea, the significant differences in internal and external rotation found in this study suggest that analysing rotational and translational parameters of joint laxity independently of one another could contribute to a deeper understanding of laxity of the knee joint, including its contributing factors, as well as the role of individual structures at different joint angles. Here, an analysis of rotational stability at full knee extension would clearly be of benefit for improving clinical understanding of joint stability, but the different ligament tensions, as well as a higher joint congruency and locking of the joint in a position of maximal stability [5, 61], prevented an analysis of the axial rotation at this position. Furthermore, such an assessment was avoided due to practical considerations, including the inevitable tensioning of the hamstrings and unavoidable rotation of the hip during application of the axial torque within our device [23]. Moreover, since a specific quantification of the contribution on stabilization of each knee ligament would be difficult in vivo, further investigation should focus on the relative contribution of the active structures on the stabilization of the knee joint as well as the parameters that influence rotational stability and are thus able to reduce the risk for ACL re-rupture. However, the results of this study provide evidence of the progressive reduction of joint rotational laxity after ACL reconstruction towards the more stable contralateral knee joint, but also that the contribution of the ACL towards joint stability is flexion dependent.
